# RFID Technology for Intraoperative Localisation of Small Colorectal Tumours: Electromagnetic Analysis and Experimental Validation

**DOI:** 10.3390/diagnostics16091318

**Published:** 2026-04-28

**Authors:** Bogdan Mocan, Mihaela Mocan, Mircea Fulea, Mircea Murar, Zsolt Mate, Adrian Calborean, Vasile Virgil Bintintan

**Affiliations:** 1 Department of Design Engineering and Robotics, Technical University of Cluj-Napoca, 400114 Cluj-Napoca, Romania; bogdan.mocan@muri.utcluj.ro (B.M.); mircea.fulea@staff.utcluj.ro (M.F.); mircea.murar@muri.utcluj.ro (M.M.); 2 Department of Internal Medicine, Iuliu Hațieganu University of Medicine and Pharmacy, 400012 Cluj-Napoca, Romania; 3 Department of Circuit Design, Tehnologistic, 407035 Cluj-Napoca, Romania; zsolt.mathe@tehnologistic.ro; 4 National Institute for Research and Development of Isotopic and Molecular Technologies, Donath Street, No 67-103, 400293 Cluj-Napoca, Romania; adrian.calborean@itim-cj.ro; 5 Department of Surgery, Iuliu Hațieganu University of Medicine and Pharmacy, 400012 Cluj-Napoca, Romania; vbintintan@gmail.com

**Keywords:** radio frequency identification (RFID), tumour localisation, minimally invasive surgery, colorectal cancer, electromagnetic simulation, near-field communication (NFC), intraoperative guidance, tissue-equivalent phantom, surgical navigation

## Abstract

**Background/Objectives**: Accurate intraoperative tumour localisation remains challenging in minimally invasive colorectal surgery, where conventional tattooing methods suffer from marker migration, tissue diffusion, and potential allergic reactions. Radio frequency identification (RFID) technology offers a promising alternative through implantable passive transponders detectable via electromagnetic coupling, eliminating ionising radiation exposure. **Methods**: This preclinical feasibility study evaluated three RFID frequency bands for surgical tumour marking: 134 kHz (low frequency, LF), 13.56 MHz (high frequency, HF), and 868 MHz (ultra-high frequency, UHF). Finite element electromagnetic simulations characterised antenna field distributions, while experimental validation employed glass-encapsulated transponders in air and tissue-simulating saline (0.9% NaCl, σ ≈ 1.5 S/m). Detection ranges were measured across 28 angular configurations with expanded measurement uncertainty (k = 2) ranging from ±0.9 to ±3.2 mm. **Results**: Maximum detection distances in air were 25.0 ± 0.9 mm (LF), 23.0 ± 1.1 mm (HF), and 68.0 ± 2.3 mm (UHF). In saline, ranges decreased to 22.5 ± 1.0 mm, 20.7 ± 1.2 mm, and 18.0 ± 1.4 mm, respectively, demonstrating tissue attenuation of 10% at LF/HF vs. 74% at UHF. Angular characterisation revealed 64–70% range reduction at orthogonal orientation for LF/HF systems. Computational–experimental correlation yielded r^2^ = 0.975 across 154 paired observations. **Conclusions**: The 13.56 MHz HF band emerges as the optimal candidate for clinical translation, offering adequate tissue penetration (20.7 mm), superior antenna miniaturisation potential (5 mm diameter), established biocompatibility pathways, and mature near-field communication ecosystem support. Future development should address angular sensitivity through multi-axis antenna configurations and validation in anatomically realistic tissue phantoms. This study establishes the electromagnetic evidence base for clinical system development; translation to clinical practice requires sequential preclinical and clinical evaluation.

## 1. Introduction

Colorectal cancer (CRC) ranks as the third most commonly diagnosed malignancy worldwide, with approximately 1.93 million new cases and a five-year prevalence of 5.25 million individuals according to GLOBOCAN 2022 estimates [1,2]. Although roughly 80% of diagnoses occur in individuals aged 55 years or older, an alarming epidemiological shift has emerged: incidence rates are increasing by 1–2% annually among adults under 50, positioning CRC as the leading cause of cancer-related mortality in men under 50 and the second leading cause in women of the same age group [3,4,5].

The treatment landscape has been transformed by minimally invasive surgical (MIS) approaches. Multiple randomised controlled trials have established that laparoscopic resection yields equivalent oncologic outcomes to open surgery while conferring reduced blood loss, shortened hospital stay, and accelerated recovery [6,7]. MIS now accounts for a growing majority of colectomy procedures, with adoption rates increasing steadily over the past decade [8]. However, minimally invasive rectal surgery presents unique challenges: pelvic dissection demands precise navigation within a narrow operative field; the absence of tactile feedback precludes manual palpation of tumour margins; and circumferential resection margin involvement remains the strongest predictor of local recurrence [9]. The increasing proportion of left-sided tumours—rectal cancer rising from 27% in 1995 to 31% in 2019 [4]—further underscores the need for reliable intraoperative localisation in this anatomical region.

Current methods for intraoperative tumour localisation each present significant limitations. Endoscopic tattooing, recommended as standard practice by both the ASGE and ESGE, achieves variable accuracy (70–100%) with transmural spillage rates up to 14.3%, risking peritonitis and disrupted dissection planes [10,11]. Near-infrared fluorescence imaging is constrained by limited tissue penetration (<10 mm), insufficient for the 15–30 mm depth spanning bowel wall and mesorectal fat [12,13,14]. Alternative guidance technologies—including electromagnetic navigation, intraoperative ultrasound, and AI-assisted computer vision—each exhibit fundamental constraints discussed in detail in Section 2.1.

Radio frequency identification (RFID) technology addresses this clinical gap through wireless electromagnetic coupling at tissue depths of 20–25 mm, preoperative marking stability over weeks, and real-time distance feedback without complex three-dimensional reconstruction [15,16,17]. Clinical viability has been established in breast surgery, where the LOCalizer system (Hologic) has achieved 100% tag retrieval rates in prospective series exceeding 500 patients [18,19,20,21,22]. However, translation to colorectal applications requires systematic investigation of frequency-dependent electromagnetic behaviour in pelvic tissues, which differ substantially from breast tissue in layered composition, dielectric properties, and surgical access geometry. A comprehensive literature review reveals no published studies evaluating RFID technology for colorectal tumour localisation.

The need to study frequency-dependent electromagnetic behaviour specifically in colorectal tissues stems from fundamental differences relative to the breast tissue environment where RFID localisation has been clinically validated. The colorectal wall comprises a four-layer dielectric structure—mucosa (εr ≈ 138, σ ≈ 0.60 S/m), submucosa, muscularis propria, and mesorectal fat (εr ≈ 12, σ ≈ 0.03 S/m)—creating a heterogeneous propagation medium with substantially greater dielectric contrast between layers compared to breast tissue, which is predominantly adipose (εr ≈ 5–12). Furthermore, the surgical access geometry in laparoscopic colorectal procedures differs fundamentally: the detection instrument approaches the transponder from the serosal surface rather than transcutaneously, must operate within a narrow pelvic operative field, and must fit within laparoscopic port dimensions (5–12 mm). These distinctions preclude direct extrapolation of breast surgery RFID performance data to colorectal applications and motivate the present tri-frequency investigation.

This paper presents a comparative evaluation of passive RFID systems operating at 134 kHz (low frequency), 13.56 MHz (high frequency), and 868 MHz (ultra-high frequency) for colorectal tumour localisation. The study addresses three objectives: (1) characterise magnetic and electromagnetic field distributions through finite element method simulation; (2) quantify detection range performance across 28 angular configurations in air and physiological saline; and (3) establish evidence-based frequency selection criteria for clinical system development.

The expected trade-offs across frequency bands can be anticipated from electromagnetic theory: low-frequency systems maximise tissue penetration due to large skin depth but require relatively large antenna structures; ultra-high-frequency systems enable the smallest transponder form factors but suffer exponential tissue attenuation; and high-frequency systems offer a potential engineering compromise between these extremes. Successful clinical implementation of RFID technology for colorectal tumour localisation could eliminate the limitations of conventional tattooing—including marker migration, tissue diffusion, and allergic reactions—while providing stable preoperative marking, real-time distance feedback during surgery, and potentially reducing re-excision rates through improved margin assessment.

## 2. Background and Related Work

### 2.1. Current Tumour Localisation Technologies in Colorectal Surgery

As introduced in Section 1, current localisation methods all present clinically significant limitations. This section provides detailed context for each technology class.

Endoscopic tattooing protocols employ sterile India ink, indocyanine green (ICG), or commercially prepared carbon-based suspensions injected submucosally at three to four quadrants adjacent to the lesion [10]. A pooled analysis reported an overall error rate of 9.5%, encompassing incorrect localisation, failed visualisation, and incomplete marking [10]. Transmural injection complications include localised peritonitis, infected haematoma, abscess formation, inflammatory pseudotumour, and adhesions [11].

Near-infrared (NIR) fluorescence tattooing has demonstrated enhanced visibility compared to standard India ink. Kakizoe and colleagues reported excellent tumour visibility without compromising anatomical plane identification [23]. ICG fluorescence navigation has additionally gained attention for anastomotic perfusion assessment and lymphatic drainage mapping, though standardised protocols for tumour localisation remain lacking [23,24]. However, the fundamental depth limitation (<10 mm penetration) restricts applicability for deeply seated lesions requiring detection through 15–30 mm of tissue.

Among alternative guidance technologies, electromagnetic navigation (EMN) achieves submillimetre tracking accuracy but suffers from ferromagnetic instrument interference and prohibitive cost (>$100,000) [25]. Intraoperative ultrasound demonstrates operator-dependent sensitivity (53–98%) and cannot localise preoperatively marked but visually occult lesions [26]. AI-assisted computer vision remains inherently surface-limited, and is unable to detect submucosal markers through intact serosa [27]. Recent evidence-based guidelines from the Society of American Gastrointestinal and Endoscopic Surgeons (SAGES, 2025) recommend ICG fluorescence imaging for intraoperative identification of primary abdominal cancers but acknowledge variability in outcomes depending on tumour type, neoadjuvant therapy, and injection protocol, underscoring the continued need for complementary localisation technologies [28].

### 2.2. RFID Frequency Band Characteristics and Tissue Interaction

RFID systems operate across three primary frequency bands with distinct electromagnetic coupling mechanisms and tissue interaction profiles [17,29].

Low-frequency (LF) RFID at 125–134.2 kHz utilises purely inductive coupling, functioning as a loosely coupled transformer [30]. The reactive near-field mechanism provides superior tissue penetration due to negligible absorption (skin depth δ > 1 m in muscle), relative insensitivity to dielectric variations, and omnidirectional detection capability. However, LF systems are constrained by limited range (<10 cm in air) and slow data transfer (≤4 kbps).

High-frequency (HF) RFID at 13.56 MHz balances tissue penetration with enhanced communication performance (≤424 kbps). The NFC standard at this frequency has stimulated interest in wearable and implantable medical devices [31]. Ferrite-core geometries enable flux concentration and miniaturisation to diameters compatible with laparoscopic channels (5–12 mm), though HF systems demonstrate greater sensitivity to metallic objects and potential detuning near surgical instruments [30].

Ultra-high-frequency (UHF) RFID at 860–960 MHz transitions from reactive near-field to radiative far-field coupling, enabling extended free-space ranges (several metres) and the smallest transponder form factors [32]. However, the skin depth at 868 MHz in muscle tissue (δ ≈ 42 mm) becomes comparable to clinically relevant detection distances, producing substantially higher absorption losses than LF/HF bands.

A comprehensive recent review by Zou et al. (2025) on NFC/RFID-enabled wearables and implants for biomedical applications confirms the growing maturity of the 13.56 MHz NFC ecosystem for implantable devices, demonstrating successful integration of wireless power transfer, sensing, and data communication in a range of subcutaneous and deep-tissue platforms [33].

### 2.3. RFID Systems in Surgical Oncology Applications

The LOCalizer system (Hologic) represents the most extensively validated RFID localisation platform, receiving FDA clearance (K171067) in 2017 for breast lesion localisation [34]. The system employs a passive 134 kHz transponder (11 × 2 mm, borosilicate glass encapsulation) deployable through a 12-gauge introducer needle, with a unique five-digit identification code enabling multi-tag differentiation [18].

Clinical evidence demonstrates robust performance: Christenhusz and colleagues reported 92.7% clear margin rates with 3.1% re-excision in a 100-patient multicentre study [16]; Lowes and colleagues achieved 100% tag retrieval across 150 patients [18,35]; and a 258-patient UK evaluation confirmed >95% localisation accuracy within the 10 mm NHS standard [22].

Alternative wireless technologies provide comparative context. The SAVI SCOUT system (Cianna Medical) employs radar-based localisation at 915 MHz with a reported 60 mm detection range, though this likely represents performance in air [19]. Magnetic seed localisation (Magseed, Endomag) offers full MRI compatibility but requires clearance of all ferrous materials during detection [36]. None of these technologies has been evaluated for colorectal applications.

A recent simulation model comparison by Sanli et al. (2024) evaluated Magseed and LOCalizer RFID in turkey breast phantoms, reporting 100% localisation success with both methods and no significant difference in surgical margins, confirming the technical equivalence of magnetic and RFID approaches in phantom settings [37]. A 2025 meta-analysis of RFID tag localisation by Daly et al., encompassing multiple prospective series, confirmed >95% localisation accuracy and high clinical acceptability across centres [19]. Furthermore, a 2026 pooled meta-analysis of 2117 patients undergoing Magseed localisation reported overall positive margin rates of 7.6% and retrieval success approaching 100%, providing an important benchmark for wireless localisation efficacy [38]. A prior feasibility study by Joo et al. (2019) evaluated RFID clip markers in an ex vivo porcine gastrointestinal model, reporting detection distances of 3.5–12.5 mm through gastric and colonic wall tissue [39]. While demonstrating proof-of-concept for GI tract RFID localisation, the limited detection ranges achieved—substantially below the 15–30 mm required for clinical colorectal application—underscore the need for optimised antenna designs and systematic frequency evaluation as pursued in the present study.

### 2.4. Electromagnetic Behaviour in Biological Tissues

Tissue electromagnetic interaction is governed by frequency-dependent dielectric properties—relative permittivity (ε_r_) and conductivity (σ). Gabriel and colleagues established foundational parametric models across 10 Hz to 20 GHz [40], subsequently expanded by the IT’IS Foundation database offering standardised values for computational modelling [41].

For passive RFID systems operating at <200 µW effective radiated power, Specific Absorption Rate (SAR) values are expected to be orders of magnitude below ICNIRP regulatory limits (2 W/kg averaged over 10 g tissue) [42], and SAR analysis is therefore not pursued in this study.

Finite element method (FEM) simulation has become the standard approach for predicting electromagnetic field distributions in biological environments [43]. Validation against experimental measurements is essential, with correlation coefficients r^2^ > 0.95 and prediction errors below 12% representing accepted benchmarks for medical device modelling.

### 2.5. Knowledge Gap for Colorectal RFID Applications

Translation from breast to colorectal RFID localisation presents specific electromagnetic challenges. The colorectal wall comprises a layered dielectric structure—mucosa (ε_r_ ≈ 138, σ ≈ 0.60 S/m), submucosa, muscularis propria, and mesorectal fat (ε_r_ ≈ 12, σ ≈ 0.03 S/m)—creating a heterogeneous propagation medium with total thickness ranging from 8 to 25 mm [41,44]. For quantitative reference, breast adipose tissue presents εr ≈ 5.4 and σ ≈ 0.05 S/m at 13.56 MHz [41], compared to colorectal mucosa (εr ≈ 138, σ ≈ 0.60 S/m)—a 25-fold permittivity difference that fundamentally alters electromagnetic coupling behaviour and precludes direct performance extrapolation. This layered anatomy differs from breast tissue in both dielectric contrast between layers and overall thickness. The heterogeneity of the colorectal wall has direct implications for electromagnetic signal propagation. The order-of-magnitude difference in relative permittivity between mucosa (εr ≈ 138) and mesorectal fat (εr ≈ 12) creates impedance discontinuities at tissue interfaces that can cause partial reflection and refraction of electromagnetic waves, particularly at UHF where the wavelength becomes comparable to layer thicknesses. At LF/HF, where the quasi-static approximation holds, these dielectric contrasts have minimal effect on magnetic field coupling; however, the conductivity gradient from mucosa (σ ≈ 0.60 S/m) through muscle to fat (σ ≈ 0.03 S/m) influences the effective attenuation along the signal path. The tissue thickness range of 8–25 mm is critically important for system design; it defines the minimum required detection distance that the RFID system must achieve, and it determines whether the skin depth at a given frequency is sufficient for adequate signal penetration. When the skin depth δ is substantially larger than the tissue thickness (as at LF/HF), attenuation is minimal; when δ approaches the tissue thickness (as at UHF, δ = 42 mm), exponential signal loss becomes clinically significant.

Surgical access geometry imposes additional constraints: the detection instrument must fit within laparoscopic port dimensions (5–12 mm), the tag is approached from the serosal rather than cutaneous surface, and metallic instruments in the operative field may interfere with electromagnetic coupling. The LOCalizer system’s 70 mm transcutaneous range, validated for breast tissue, provides no direct indication of laparoscopic detection performance through the bowel wall.

Transcutaneous validation data from breast surgery are insufficient for predicting laparoscopic colorectal performance for several reasons: (1) the tissue composition differs fundamentally—breast tissue is predominantly adipose (low conductivity and low permittivity), whereas colorectal tissue presents high-conductivity mucosal and muscular layers; (2) the surgical approach geometry is reversed—breast surgery approaches from the skin surface through relatively homogeneous fat, while laparoscopic colorectal surgery approaches from the serous surface through the full bowel wall thickness; and (3) the operative environment introduces metallic laparoscopic instruments that may cause electromagnetic interference absent in breast surgery. These factors collectively necessitate independent tri-frequency characterisation specific to the colorectal application, as presented in this study.

The present study addresses this knowledge gap through computational electromagnetic simulation, tissue phantom experimentation, and systematic tri-frequency performance characterisation, establishing the evidence base necessary for clinical system development.

## 3. Materials and Methods

This study involved exclusively computational electromagnetic simulations and experimental measurements using tissue-simulating saline phantoms. No human subjects, human tissue samples, or animal subjects were involved in any phase of the research (see Institutional Review Board Statement).

### 3.1. RFID System Configurations

The investigation evaluated passive RFID transponders across three frequency bands using representative commercial components selected for each regime (Table 1).

Low-frequency (134 kHz) system: The LF transponder was an EM4100-compatible glass-encapsulated tag (Ø2 × 12 mm) incorporating an air-core solenoid antenna and a read-only integrated circuit operating at 134 kHz via inductive coupling. The glass capsule provides a biocompatible form factor analogous to commercially available surgical RFID tags such as the LOCalizer (Hologic, Marlborough, MA, USA), which employs a similar architecture validated for breast lesion localisation [18,19]. The LF reader comprised a custom microcontroller-based (MCU) platform driving an air-core cylindrical solenoid antenna (Ø10 × 10 mm, ~1000 turns, AWG 40 wire), generating an unmodulated magnetic interrogation field. The air-core cylindrical solenoid geometry was selected for LF because it generates a purely axial magnetic field with dipole topology, providing well-characterised field distributions amenable to analytical validation via the Biot–Savart law. The antenna dimensions (Ø10 × 10 mm) directly influence the magnetic field intensity through the relationship between turn count, excitation current, and winding geometry (N·I/L), with larger diameters producing stronger but more rapidly decaying axial fields.

High-frequency (13.56 MHz) system: The HF transponder was an NTAG216 (NXP Semiconductors, Eindhoven, The Netherlands) glass-encapsulated tag (Ø2 × 12 mm) compliant with the NFC Forum Type 2 Tag specification and ISO 14443A standard. The glass capsule enclosure matches the LF transponder form factor, enabling direct cross-frequency comparison under identical geometric conditions. HF interrogation was performed using the ST STEVAL-25R3916B evaluation board (STMicroelectronics, Schiphol, The Netherlands), based on the ST25R3916B NFC reader IC with configurable output power and receiver sensitivity enabling detection at field strengths below 0.15 A/m [45]. The reader antenna comprised an open NiZn ferrite rod (Ø5 × 15 mm, μ_i_ = 125) with helical winding (10 turns, 0.3 mm wire), selected for its flux concentration capability and form factor compatibility with laparoscopic instrument channels. Compliance with the NFC Forum and ISO 14443A standards ensures interoperability with the extensive NFC reader ecosystem, provides access to certified reader ICs with integrated analogue front ends, and establishes regulatory precedent for implantable NFC devices, reducing development risk for clinical translation. The ST25R3916B achieves this low detection threshold through an integrated low-noise receiver with automatic gain control and adaptive noise suppression, enabling an extended detection range through receiver sensitivity enhancement rather than increased transmitted power.

Ultra-high-frequency (868 MHz) system: The UHF transponder was a Murata LXMS21ACNA ceramic chip module (2.0 × 1.25 mm), integrating a miniaturised antenna and UHF RFID IC in a surface-mount package operating within the European ETSI band (865–868 MHz). UHF interrogation employed a commercial Impinj Speedway R420 reader operating at 868 MHz with configurable output power (10–30 dBm) and a 60 × 60 mm near-field loop antenna designed for reactive near-field coupling [34,41,42].

Table 1 summarises the complete system configurations. All transponders were passive devices deriving operational power exclusively from the interrogating electromagnetic field.

### 3.2. Computational Electromagnetic Modelling

The five-order-of-magnitude frequency span from 134 kHz to 868 MHz necessitates distinct simulation methodologies: magneto-quasi-static (MQS) analysis for LF/HF bands where wavelength vastly exceeds system dimensions, and full-wave electromagnetic simulation for UHF where wavelength becomes comparable to antenna and separation dimensions. The finite element method (FEM) was selected as the computational framework because it enables prediction of electromagnetic field distributions and detection range performance across arbitrary geometries and material configurations without requiring physical prototyping of each antenna–transponder–tissue combination—a critical advantage when exploring the three-frequency design space with multiple antenna geometries. The wide frequency range necessitates fundamentally different simulation approaches because the ratio of system dimensions to electromagnetic wavelength (L/λ) determines whether the quasi-static approximation is valid: at LF/HF where L/λ << 0.1, magnetic and electric fields are decoupled, enabling efficient MQS formulation; at UHF where L/λ ≈ 0.2, coupled electric and magnetic field propagation requires the full Maxwell equations.

#### 3.2.1. Theoretical Framework

Quasi-static approximation (LF/HF): At 134 kHz and 13.56 MHz, electromagnetic wavelengths (λ ≈ 2.24 km and 22.1 m, respectively) vastly exceed all relevant system dimensions. For the largest dimension in this study (~70 mm reader–tag separation), L/λ equals 3.1 × 10^−5^ at 134 kHz and 3.2 × 10^−3^ at 13.56 MHz—both well below the 0.1 threshold for quasi-static validity [46,47,48]. Under the MQS approximation, the magnetic vector potential $\vec{A}$ satisfies

(1) $$\nabla \times \left(\vartheta \nabla \times \vec{A}\right)+j\omega \sigma \vec{A}=\vec{J_{s}}$$

where ν = 1/μ denotes magnetic reluctivity, σ is electrical conductivity, ω = 2πf is the angular frequency, and $\vec{Js}$ is the source current density. The magnetic flux density is obtained from $\vec{B}$ = ∇ × $\vec{A}$. For axisymmetric geometries, the problem reduces to a two-dimensional formulation in the (r, z) plane as follows:

(2) $$\frac{\partial }{\partial r}\left[\vartheta \cdot \frac{1}{r}\frac{\partial \left(rA_{\varphi }\right)}{\partial r}\;\right]+\frac{\partial }{\partial z}\left[\vartheta \cdot \frac{\partial A_{\varphi }}{\partial z}\right]+j\omega \sigma A_{\varphi }=J_{s}$$

Full-wave formulation (UHF): At 868 MHz (λ ≈ 345 mm), the wavelength becomes comparable to antenna dimensions and reader–tag separation, necessitating full-wave analysis. The governing time-harmonic vector wave equation [49] is

(3) $$\nabla \times \left(\mu r^{-1}\nabla \times \vec{E}\right)-k_{0}^{2}\varepsilon c\vec{E}=0$$

where k_0_ = ω√(ε_0_μ_0_) is the free-space wavenumber and ε_c_ = ε_r_ − jσ/(ωε_0_) is the complex relative permittivity incorporating both dielectric and conductive losses. At UHF in biological tissues, both components contribute substantially to attenuation, unlike the predominantly conductive losses at LF/HF. The magnetic field is obtained from Faraday’s law

(4) $$\vec{H}={\left(j\omega \mu_{0}\mu_{r}\right)}^{-1}\cdot \nabla \times \vec{E}$$

#### 3.2.2. Low Frequency (134 kHz)

Simulations employed FEMM 4.2, an open-source 2D finite element solver for low-frequency magnetic field problems [50,51]. Table 2 summarises solver configurations across all three frequency bands.

Antenna geometry model. The air-core solenoid reader antenna (Ø10 × 10 mm, 1000 turns AWG 40, Table 3) was modelled as an equivalent current sheet with uniform density distributed across the winding cross-section—a homogenisation valid when wire diameter is small relative to coil dimensions [52]. The source current density was calculated as

(5) $$J_{s}=\frac{N\cdot I}{A_{wind}}=\frac{1000\times 0.1}{2.5\times 10}\times \frac{1}{k_{p}}\approx 6.2\;A/{mm}^{2}$$

where N is the turn count, I = 100 mA peak excitation current, A_wind_ is the geometric winding cross-section, and k_p_ ≈ 0.65 is the packing factor for multi-layer random-wound coils with AWG 40 wire.

Mesh and boundary conditions: Adaptive mesh refinement concentrated elements in regions of high field gradient: coil region (h_max_ = 0.2 mm, resolving δ_Cu_ ≈ 0.18 mm skin depth in copper), near-field region 0–30 mm (h_max_ = 0.5 mm), far-field 30–200 mm (graded to 5 mm), and tissue/saline regions (h_max_ = 1.0 mm with interface refinement). Mesh independence was verified with <1% field variation upon further refinement. The computational domain extended to 200 mm radially and ±150 mm axially with asymptotic boundary conditions enforcing r^−3^ dipole field decay [53]. Interface conditions enforced continuity of tangential $\vec{H}$ and normal $\vec{B}$ at material boundaries. The fine mesh requirement in the coil region (hmax = 0.2 mm) arises directly from the electromagnetic skin depth in copper at 134 kHz, δCu = √(2/ωμσ) ≈ 0.18 mm. Accurate resolution of the current distribution within the conductor requires at least 2–3 elements through the skin depth thickness, dictating this mesh constraint. Conversely, in the far-field region where the magnetic field decays as r^−3^, the field gradient diminishes rapidly, permitting progressive mesh coarsening (grading from 0.5 mm to 5 mm) that substantially reduces computational cost—from a potential >10^6^ elements to 45–65 k elements—without compromising solution accuracy at the clinically relevant detection threshold locations.

Material properties were assigned as per Table 4, with tissue values from the IT’IS Foundation database [54,55]. At 134 kHz, displacement current (ωε_0_ε_r_) is negligible compared to conduction current (σ) for all materials, confirming MQS validity. Quantitatively, the ratio ωε_0_εr/σ evaluates to approximately 4 × 10^−4^ for physiological saline (εr = 80, σ = 1.5 S/m) and <10^−3^ for all tissue types at this frequency, confirming that displacement current contributes less than 0.1% to total current density and validating the magneto-quasi-static approximation. At 868 MHz, however, this ratio approaches unity for high-water-content tissues, necessitating the full-wave formulation that retains both current components.

#### 3.2.3. High Frequency (13.56 MHz)

Simulations employed COMSOL Multiphysics 6.2 with the AC/DC Module (Table 2), selected for robust handling of complex permeability tensors essential for ferrite modelling [52].

Ferrite-core antenna geometries: Three configurations were modelled to evaluate trade-offs between detection range, directionality, and surgical probe compatibility (Table 5): (a) an open ferrite rod (NiZn, Ø5 × 15 mm, and 10-turn helical winding) producing bidirectional axial emission; (b) a shielded variant with 0.5 mm copper rear hemisphere redirecting flux forward; and (c) a pot core (Ø10 × 5 mm, 15-turn central winding) providing high near-field concentration with rapid spatial decay [56,57,58].

Ferrite material model: NiZn ferrite was selected based on high resistivity (>10^5^ Ω·m), moderate initial permeability (μ_i_ = 125), and stable high-frequency performance [36]. Complex permeability is modelled as

(6) $$\mu =\mu^{\prime }(1-jtan\delta_{\mu })$$

with μ′ ≈ 120 and tan δ_μ_ ≈ 0.02 at 13.56 MHz. The effective permeability of the rod antenna is reduced by demagnetisation as follows:

(7) $$\mu_{eff}=\frac{\mu_{r}}{1}+N(\mu_{r}-1)$$

where the demagnetisation factor N ≈ 0.1 for the 3:1 aspect ratio rod yields μ_eff_ ≈ 10.7 [46].

Material properties at 13.56 MHz incorporate both conductive and dielectric tissue contributions (Table 6). Mesh refinement followed COMSOL’s physics-controlled ‘Finer’ preset with manual enhancement: ferrite core (h_max_ = 0.3 mm), winding region (h_max_ = 0.1 mm with 3-layer boundary mesh for δ_Cu_ ≈ 18 µm), and near-field (h_max_ = 0.8 mm). The mesh independence criterion was <2% variation at the detection threshold distance.

#### 3.2.4. Ultra-High Frequency (868 MHz)

Full-wave simulations employed COMSOL 6.2 with the RF Module (Table 2), solving the complete vector wave equation [51].

Near-field UHF antenna: The reader antenna was modelled as a segmented loop (4 segments, 60 × 60 mm, Table 7) on FR-4 substrate, designed to concentrate the magnetic field in the reactive near-field region while minimising far-field radiation [50].

Transponder model: The Murata LXMS21ACNA chip module was modelled using the manufacturer-specified equivalent circuit parameters [50]. The tag activation threshold was set at Pth = −20 dBm (10 µW). Detection was defined as the reader–tag separation at which delivered IC power equals or exceeds this threshold as follows:

(8) $$P_{IC}=\frac{1}{2}{\left|V_{IC}\right|}^{2}R_{e}\{Y_{IC}\}$$

Tissue model: At 868 MHz, accurate modelling requires frequency-dependent complex permittivity for each tissue layer (Table 8, IT’IS Foundation [41]). The skin depth δ = √(2/ωμσ) ranges from 40 to 48 mm for high-water-content tissues (muscle or mucosa) to 220 mm for fat, indicating significant UHF attenuation within typical tissue thicknesses.

Domain truncation and mesh: A spherical perfectly matched layer (PML) of thickness λ/4 ≈ 86 mm with quadratic grading (reflection coefficient < −40 dB) terminated the computational domain [51,52,53]. Element size satisfied h_max_ ≤ λ/(10√ε_r_,max), yielding h_max_ ≤ 3.9 mm in tissue regions (ε_r_ = 78). The complete 3D model required 1.5–2.5 million tetrahedral elements, 32 GB of RAM, and 12–16 h on a mobile workstation (Intel Core i9-13900HX (Santa Clara, CA, USA), 16 cores/24 threads, 64 GB DDR5). Adaptive refinement continued until field convergence within 3%. This criterion ensures at least 10 elements per effective wavelength (λeff = λ/√εr = 345/√78 ≈ 39 mm) within the highest-permittivity medium, providing adequate spatial sampling for accurate field resolution. Computational resource requirements scale approximately as O(N^3^) with mesh density for direct solvers, explaining the substantially higher cost of UHF simulations compared to the 2D axisymmetric LF/HF models.

#### 3.2.5. Model Validation

Simulation accuracy was validated through multiple approaches:

• Analytical benchmarking: For the 134 kHz air-core solenoid in free space, computed axial field values were compared against the analytical Biot–Savart solution for a finite solenoid, with agreement within 2% at distances exceeding one coil diameter. The Biot–Savart law provides an exact analytical solution for the magnetic field of a finite solenoid in free space, serving as an absolute reference unaffected by numerical discretisation errors; agreement within 2% therefore confirms the correctness of the FEM implementation, mesh adequacy, and boundary condition formulation.
• Mesh independence: Progressive mesh refinement studies verified solution convergence, with acceptance criterion of <2% variation in field magnitude at the detection threshold distance.
• Energy conservation: Global energy balance verification confirmed that input power equals the sum of dissipated power (ohmic losses) and radiated power (for UHF) to within numerical precision.
• Experimental correlation: Simulated detection range predictions were compared against measured values across 154 paired observations (frequency, angle, and medium), with frequency-stratified correlation coefficients r^2^ = 0.973 (134 kHz), 0.969 (13.56 MHz), and 0.968 (868 MHz). Root mean square errors ranged from 1.00 mm (LF) to 3.43 mm (UHF), with maximum relative deviations bounded within ±8% (LF), ±10% (HF), and ±12% (UHF). Quantitative validation metrics including correlation analysis and Bland–Altman agreement are presented in Section 4.5.

### 3.3. Experimental Protocol

Test Apparatus

**Figure 1:**
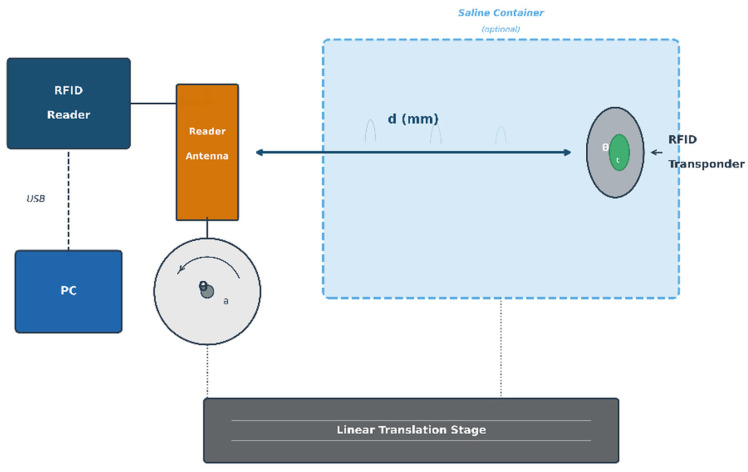
Experimental setup schematic showing precision positioning stage, reader antenna, RFID transponder, and optional saline container for tissue-equivalent measurements.

**Figure 2:**
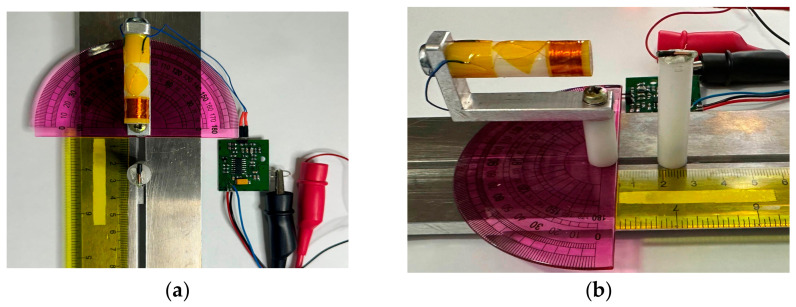
Experimental test apparatus for RFID detection range characterisation: (**a**) overall assembly showing aluminium frame structure, manual positioning mechanism, and reader electronics; (**b**) detail of antenna/transponder mounting configuration with acrylic and PTFE components in the measurement region.

A custom test apparatus was built to enable controlled positioning of both reader antenna and RFID transponder with adjustable angular and translational degrees of freedom (Figure 1 and Figure 2). The fixture employs a non-ferromagnetic aluminium frame for structural rigidity, with acrylic and PTFE mounting components in the immediate measurement region to minimise electromagnetic interference. Manual positioning is achieved through: (1) linear translation along the measurement axis, with distance measured using a graduated scale (0.5 mm divisions, estimated reading accuracy ±0.5 mm); (2) reader antenna rotation about its longitudinal axis in 30° increments spanning ±90°, set using a protractor gauge; and (3) transponder rotation about an axis perpendicular to the measurement direction in 30° increments from 0° (coaxial) to 90° (perpendicular).

Angular Configuration Matrix

Measurements were performed across a standardised angular matrix: reader antenna angles θ_a_ ∈ {−90°, −60°, −30°, 0°, +30°, +60°, +90°} and transponder angles θ_t_ ∈ {0°, 30°, 60°, 90°}, yielding 28 geometric configurations per frequency band. Exploiting the circular symmetry of the cylindrical antenna geometry, measurements at selected angular combinations were considered representative of the full three-dimensional detection envelope. All configurations were evaluated in two propagation media: air and physiological saline.

Tissue-Simulating Medium

Physiological saline solution (0.9% NaCl, σ ≈ 1.5 S/m at 25 °C) was selected as the tissue-simulating medium based on three considerations: (1) its conductivity approximates the volume-weighted average of colorectal tissue layers, providing a conservative estimate of near-field attenuation; (2) saline constitutes a reproducible, standardised medium consistent with ANSI/AAMI/ISO 14708-3 [58] methodology for implantable device testing; and (3) the homogeneous medium represents an upper-bound attenuation scenario compared to the actual layered anatomy, where low-conductivity fat layers (σ ≈ 0.02–0.05 S/m) would reduce overall signal loss. The limitations of this simplified phantom model are discussed in Section 5.4. The characterisation of saline as a conservative (upper-bound) estimate is justified quantitatively: the saline conductivity (1.5 S/m) exceeds the volume-weighted average conductivity of the colorectal wall, where the substantial contribution of low-conductivity mesorectal fat (σ ≈ 0.03 S/m) reduces the effective tissue conductivity to approximately 0.3–0.5 S/m depending on the relative proportion of fat. Consequently, actual through-tissue detection ranges are expected to equal or exceed the saline measurements reported herein.

Measurement Protocol and Detection Criterion

Detection range measurements were performed under standard laboratory conditions (22 ± 2 °C, 45 ± 10% RH). For each of the 28 angular configurations, the transponder was positioned at increasing distances from the antenna face in 0.5 mm increments until detection failure. The maximum detection distance was recorded as the last position at which valid packet reception—defined as successful protocol acknowledgment with correct identifier readback—occurred consistently across five consecutive interrogation cycles. Each angular configuration was measured in a single session per frequency band and propagation medium.

Measurement Uncertainty

The measurement uncertainty is dominated by two components: positioning stage accuracy and step resolution. The expanded uncertainty (k = 2, 95% confidence level) was calculated according to:

(9) $$U=k\times \sqrt{u_{stage}^{2}+u_{resolution}^{2}}$$

where u_stage_ = 0.5/√3 ≈ 0.29 mm (rectangular distribution for ±0.5 mm accuracy specification) and u_resolution_ = 0.5/2 = 0.25 mm (half-division visual interpolation on the graduated scale). This yields a combined standard uncertainty of u_c_ = 0.38 mm and an expanded uncertainty of U ≈ ±0.8 mm (k = 2). When combined with threshold transition uncertainty (0.3–1.2 mm depending on frequency), total expanded uncertainties range from ±0.9 mm (LF) to ±3.2 mm (UHF at oblique angles).

An additional systematic uncertainty arises from the binary nature of the detection criterion: the transition from reliable to failed detection occurs over a finite spatial interval rather than at a sharp boundary. This threshold transition width was estimated at 0.5–1.5 mm depending on frequency band and angular configuration, with broader transitions observed at UHF due to backscatter communication sensitivity to multipath reflections and impedance variations. The reported detection distances therefore represent a conservative lower bound, corresponding to the last position of consistent five-cycle detection rather than the first position of intermittent failure.

### 3.4. Statistical Analysis

All statistical analyses were performed using MATLAB R2024a to characterise agreement between computational predictions and experimental measurements and to quantify measurement uncertainty. Linear regression analysis (Pearson correlation) was used to assess the overall correspondence between FEM-predicted and experimentally measured detection ranges across 154 paired observations, with frequency-stratified analysis to identify band-specific accuracy. Correlation coefficients (r^2^), regression slopes with 95% confidence intervals, and root mean square errors (RMSEs) were calculated to quantify predictive accuracy. Bland–Altman analysis was employed as a complementary method to assess agreement without the implicit linearity assumption of correlation analysis, providing mean bias and 95% limits of agreement (LoA = mean ± 1.96 SD) for each frequency band. Measurement uncertainties were calculated following the Guide to the Expression of Uncertainty in Measurement (GUM) framework, with expanded uncertainties reported at the k = 2 confidence level (95% coverage probability). Combined standard uncertainties were propagated from individual uncertainty components (positioning stage accuracy, scale resolution, threshold transition width) using the root sum of squares method (Equation (9)). Mesh convergence was verified using a <2% variation criterion in field magnitude at the detection threshold distance upon successive mesh refinement. All correlation and regression analyses used standard least squares methods; *p*-values < 0.001 were considered statistically significant.

## 4. Results

### 4.1. Low-Frequency (134 kHz) Characterisation

**Figure 3:**
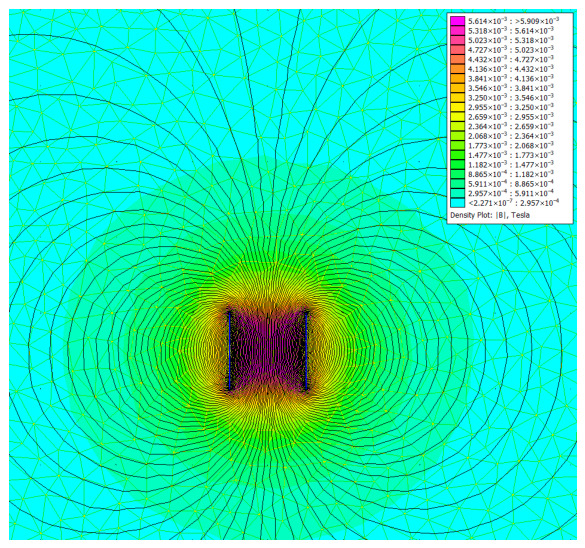
Simulated magnetic flux density distribution for 134 kHz air-core cylindrical antenna (Ø10 × 10 mm, 1000 turns).

Figure 3 illustrates the simulated magnetic flux density distribution for the 134 kHz air-core cylindrical antenna. The axisymmetric field pattern exhibits characteristic dipole topology with maximum intensity along the coil axis and r^−3^ decay in the far-field region. The calculated skin depth of 1.12 m at 134 kHz in physiological saline confirms minimal resistive absorption within all clinically relevant detection distances.

Supplementary Table S1 presents the complete detection range measurements at 134 kHz across all 28 angular configurations in both media. A maximum detection distance of 25.0 ± 0.9 mm (expanded uncertainty, k = 2) was achieved with coaxial alignment (θ_a_ = 0°, θ_t_ = 0°) in air. In physiological saline, the reference coaxial configuration yielded 22.5 ± 1.0 mm, representing a 10.0% reduction from the air baseline. The expanded uncertainties incorporate contributions from positioning stage accuracy, step resolution, and the binary detection threshold transition width, the latter varying from approximately 0.5 mm at coaxial alignment to 1.0 mm at oblique configurations where the coupling gradient steepens (see Section 3.3). Detection was achieved across all 28 angular configurations in air but failed at 2 of 28 configurations in saline (both at θ_t_ = 90° with |θ_a_| ≥ 60°). The dipole field topology arises directly from the cylindrical symmetry of the solenoid geometry; contributions from all coil turns sum constructively along the coil axis, producing maximum field intensity in this direction, while cancellation effects at off-axis positions produce the characteristic dipole pattern. At this skin depth, the exponential attenuation factor e^−2z/δ^ evaluates to 0.956 at z = 25 mm (the maximum detection distance), corresponding to only a 4.4% power loss—confirming that tissue-induced signal attenuation is negligible at LF and the 10% range reduction observed experimentally is attributable primarily to reactive near-field detuning effects rather than absorptive losses. The expanded uncertainties (k = 2) were determined following the GUM framework described in Section 3.4, combining positioning-stage accuracy (±0.5 mm rectangular distribution), scale resolution (0.5 mm divisions with visual interpolation), and the binary detection threshold transition width. The latter component varies from approximately 0.5 mm at coaxial alignment to 1.0 mm at oblique configurations, where the steeper coupling coefficient gradient (∂M/∂z increases as the mutual inductance approaches the null zone) means that small positional changes produce larger fractional changes in received-signal strength, broadening the spatial transition from reliable to failed detection.

### 4.2. High-Frequency (13.56 MHz) Characterisation

**Figure 4:**
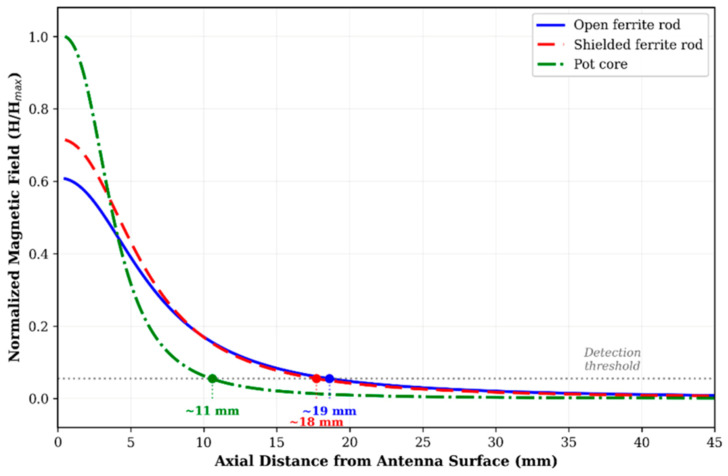
Comparative axial magnetic field decay profiles for open ferrite rod, shielded ferrite rod, and pot core antenna configurations at 13.56 MHz.

Three ferrite-core antenna geometries were compared at 13.56 MHz. Figure 4 presents the comparative field decay profiles, demonstrating distinct trade-offs between range, directionality, and form factor.

The open ferrite rod configuration was selected for subsequent angular characterisation based on optimal range-form factor balance. At coaxial alignment, the open rod achieves the longest lateral detection range (~19 mm to the 0.15 A/m threshold), while the pot core provides highest near-field intensity but more rapid spatial decay, limiting lateral range to ~11 mm. The shielded rod demonstrates intermediate performance with enhanced front-to-back ratio (>15 dB).

Comprehensive angular characterisation of the open ferrite rod antenna (Supplementary Table S1) yielded a maximum detection distance of 23.0 ± 1.1 mm in air at coaxial alignment (θ_a_ = 0°, θ_t_ = 0°), representing an 8.0% reduction vs. the 134 kHz system. Saline measurements at the same configuration yielded 20.7 ± 1.2 mm—a 10.0% tissue attenuation consistent with LF observations, confirming that the skin depth at 13.56 MHz (δ = 111 mm) remains substantially larger than detection distances, maintaining operation in the low-attenuation regime. The angular null zone at θ_t_ = 90° produced detection ranges between 5.5 and 8.2 mm in air, representing a 64–76% reduction from the coaxial maximum, with complete detection failure at 3 of 28 saline configurations (θ_t_ = 90°, |θ_a_| ≥ 30°). This severe range reduction at orthogonal transponder orientation is a direct consequence of the cos(θ) dependence of mutual inductance between coaxial coils; as the transponder axis rotates to 90° relative to the reader field, the magnetic flux linkage through the transponder coil approaches zero, producing a theoretical coupling null. The non-zero measured values (5.5–8.2 mm) arise from secondary coupling mechanisms including electric field coupling, non-ideal coil geometry effects, and ferrite core flux spreading. Detection failure at 3 of 28 saline configurations (θt = 90°, |θa| ≥ 30°) occurs where this reduced coupling, combined with saline-induced attenuation, decreases the received signal below the transponder IC activation threshold.

### 4.3. Ultra-High-Frequency (868 MHz) Characterisation

**Figure 5:**
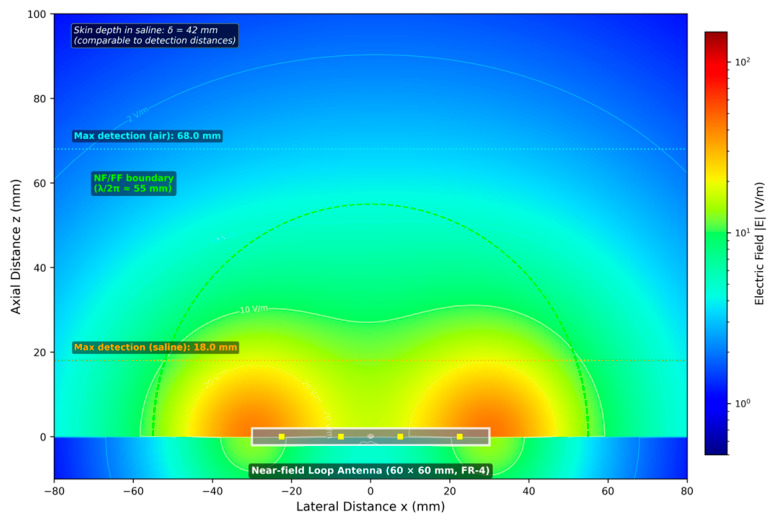
Simulated electric field distribution for near-field UHF antenna (60 × 60 mm) at 868 MHz.

Figure 5 illustrates the simulated electric field distribution for the near-field UHF antenna. Unlike LF/HF systems, UHF propagation exhibits coupled electric and magnetic field components with significant radiative character. The field pattern shows forward radiation with near-field concentration, transitioning from reactive near-field to radiating far-field behaviour at a boundary distance of approximately λ/2π ≈ 55 mm.

The maximum detection distance in air reached 68.0 ± 2.3 mm at coaxial alignment, substantially exceeding LF/HF performance by a factor of 2.7× (Supplementary Table S1). The larger expanded uncertainty at UHF reflects both the broader threshold transition width (~1.5 mm, attributed to backscatter communication sensitivity to multipath reflections and impedance variations) and the increased positioning sensitivity at extended detection distances.

However, saline immersion dramatically reversed this performance advantage: maximum detection range reduced to only 18.0 ± 1.4 mm, representing 73.5% attenuation—more than seven times the tissue-induced loss observed at LF/HF. This attenuation results from two compounding mechanisms: absorptive loss within the conducting medium (skin depth δ = 42 mm, comparable to detection distances) and reflective loss at the air–saline interface (|Γ|^2^ ≈ 0.6). Angular sensitivity was most pronounced at UHF, with the orthogonal null zone (θt = 90°) reducing detection to 14.0 ± 1.8 mm in air (79% reduction) and producing complete detection failure at 8 of 28 saline configurations. At UHF, multipath reflections from the saline container boundaries and impedance variations at the air–saline interface compound the absorptive losses, producing broader and less predictable detection threshold transitions (~1.5 mm width vs. ~0.5 mm at LF) and contributing to the larger expanded uncertainties. The 79% range reduction at orthogonal alignment (θt = 90°)—the most severe among the three bands—reflects the combined effect of magnetic coupling nulling (common to all bands) and the additional electric field polarisation sensitivity specific to UHF radiative propagation. Complete detection failure at 8 of 28 saline configurations demonstrates that UHF tissue attenuation preferentially eliminates marginal detection scenarios, severely constraining the practical angular acceptance window.

### 4.4. Cross-Frequency Comparison

**Figure 6:**
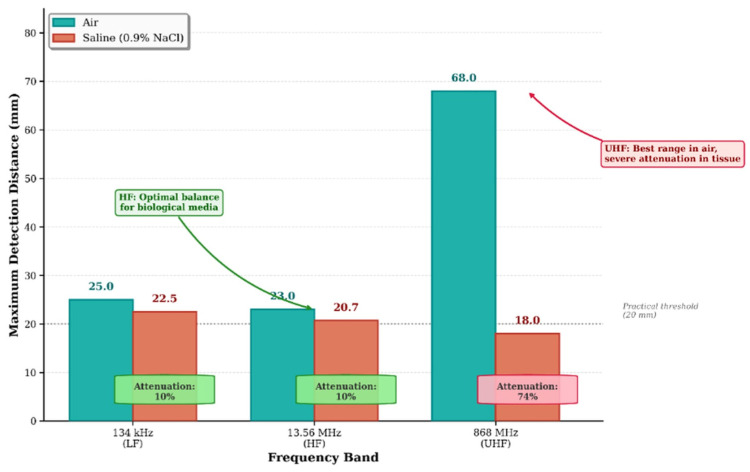
Cross-frequency detection range comparison in air vs. saline (0.9% NaCl).

Figure 6 consolidates detection range performance across all three frequency bands, highlighting the critical divergence between air and tissue-equivalent media. Despite superior air performance, UHF exhibits 74% tissue attenuation resulting in inferior through-tissue detection compared to LF (10% attenuation) and HF (10% attenuation).

**Figure 7:**
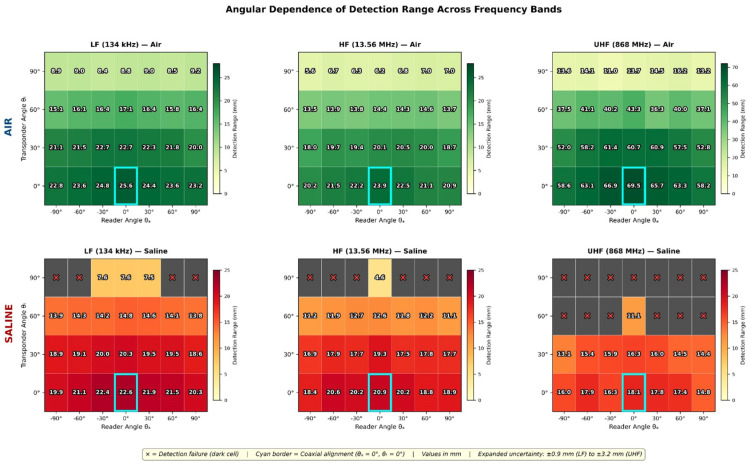
Angular dependence of detection range across frequency bands.

Figure 7 presents angular dependence heatmaps for all three frequencies, revealing the characteristic null zones at orthogonal transponder orientation (θ_t_ = 90°) where 60–79% range reduction occurs.

Table 9 summarises comprehensive performance metrics enabling evidence-based frequency selection.

Complete detection range measurements across all 28 angular configurations, three frequency bands, and both propagation media are provided in Supplementary Table S1.

### 4.5. Computational Model Validation

Quantitative validation of the computational models was performed by systematic comparison of FEM predictions against experimental measurements. A total of 154 paired observations were analysed—56 pairs at 134 kHz, 56 at 13.56 MHz, and 42 at 868 MHz—each evaluated in both air and physiological saline. The reduced count at 868 MHz reflects configurations where neither simulation nor experiment achieved detectable signal levels in saline.

**Figure 8:**
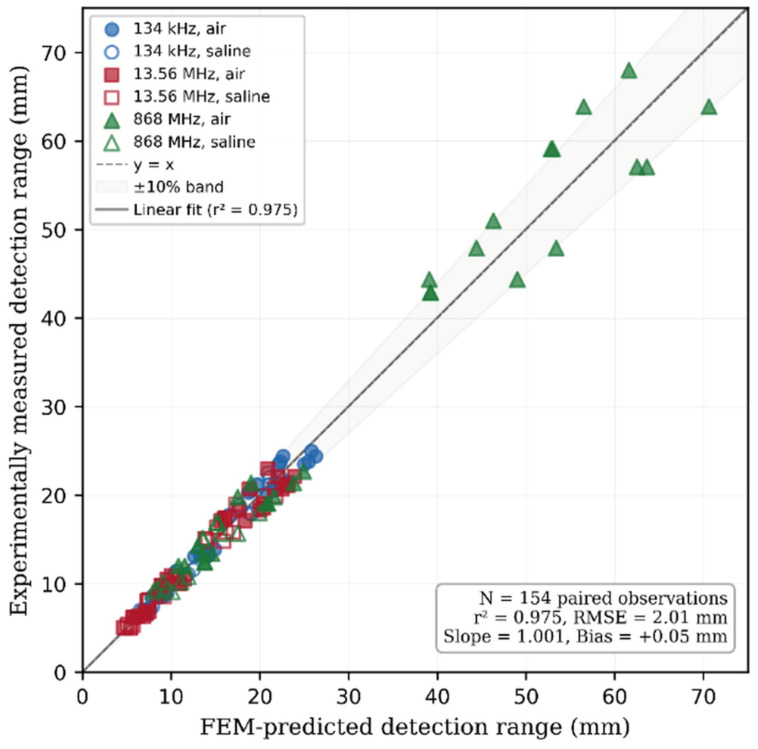
Correlation between FEM-predicted and experimentally measured detection ranges. The dashed diagonal line represents perfect agreement (y = x); the shaded band denotes ±10% deviation. Filled symbols: air measurements; open symbols: saline measurements. Each data point corresponds to a unique (frequency, angular configuration, or medium) combination. *N* = 154 paired observations.

Figure 8 presents the correlation between FEM-predicted and experimentally measured detection ranges. Linear regression across all 154 paired observations yielded r^2^ = 0.975 (*p* < 0.001) with slope 1.001 ± 0.025 and intercept 0.02 ± 0.56 mm (Table 10). The near-unity slope and intercept consistent with zero confirm the absence of systematic proportional or offset bias. The overall RMSE was 2.01 mm, and 92% of data points fell within the ±10% agreement band.

Stratification by frequency band revealed progressively decreasing agreement with increasing operating frequency (Table 10). At 134 kHz, quasi-static models achieved r^2^ = 0.973 with RMSE = 1.00 mm and maximum relative error of 8.2%, reflecting the well-characterised analytical solutions available for low-frequency solenoid fields. At 13.56 MHz, models yielded r^2^ = 0.969 with RMSE = 1.13 mm and a maximum relative error of 10.0%, the modest degradation attributable to ferrite material property uncertainty and increased sensitivity to geometric tolerances. At 868 MHz, full-wave simulations achieved r^2^ = 0.968 with RMSE = 3.43 mm and a maximum relative error of 12.1%, consistent with the greater electromagnetic complexity including coupled E/H field components, radiation effects, and heightened sensitivity to dielectric property variations.

**Figure 9:**
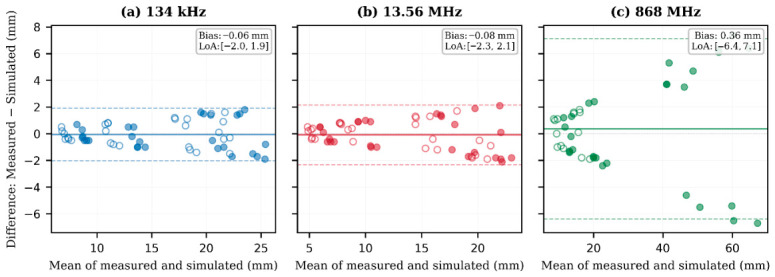
Bland–Altman agreement plots for (**a**) 134 kHz, (**b**) 13.56 MHz, and (**c**) 868 MHz. Solid horizontal lines indicate mean bias; dashed lines represent 95% limits of agreement (mean ± 1.96 SD). Filled symbols: air; open symbols: saline.

Bland–Altman analysis (Figure 9) provides complementary assessment of agreement without the implicit linearity assumption of correlation analysis. For the LF and HF bands, the mean bias was negligible (−0.06 and −0.08 mm, respectively) with narrow 95% limits of agreement—[−2.03, +1.91] mm at 134 kHz and [−2.31, +2.15] mm at 13.56 MHz—intervals substantially smaller than the 0.5 mm positioning stage resolution, confirming that model prediction uncertainty falls below the experimental measurement floor. At 868 MHz, the mean bias remained small (+0.36 mm) but LoA widened to [−6.40, +7.12] mm, reflecting the heteroscedastic error structure inherent in full-wave modelling across the large dynamic range (9–68 mm) of UHF propagation.

Two systematic error patterns emerge. First, prediction errors increase monotonically with frequency, consistent with the progressive transition from analytically tractable quasi-static fields to full-wave solutions requiring simultaneous resolution of coupled field components. Second, within each band, larger discrepancies occur at oblique configurations (θ_t_ ≥ 60°), where the steep coupling coefficient gradient amplifies sensitivity to geometric tolerances and mesh discretisation. In saline, additional error arises from conductivity uncertainty (σ ≈ 1.5 ± 0.1 S/m), which propagates through the exponential attenuation relationship with amplified effect at higher frequencies.

The overall agreement—r^2^ = 0.975, mean relative error 7.3%, maximum deviation ±12.1%, and near-unity regression slope without systematic bias—validates the FEM methodology as a reliable predictive framework for RFID detection range estimation in biological tissue environments. The frequency-dependent error structure provides a quantified uncertainty budget applicable to safety margins in clinical system design.

## 5. Discussion

### 5.1. Frequency Selection for Clinical System Development

#### 5.1.1. Fundamental Trade-Offs Across Frequency Bands

The experimental findings provide clear evidence-based guidance for frequency selection in surgical RFID localisation systems. While UHF technology offers compelling advantages in free-space performance and transponder miniaturisation, the 74% tissue attenuation fundamentally compromises its utility for through-tissue detection of implanted markers.

**Figure 10:**
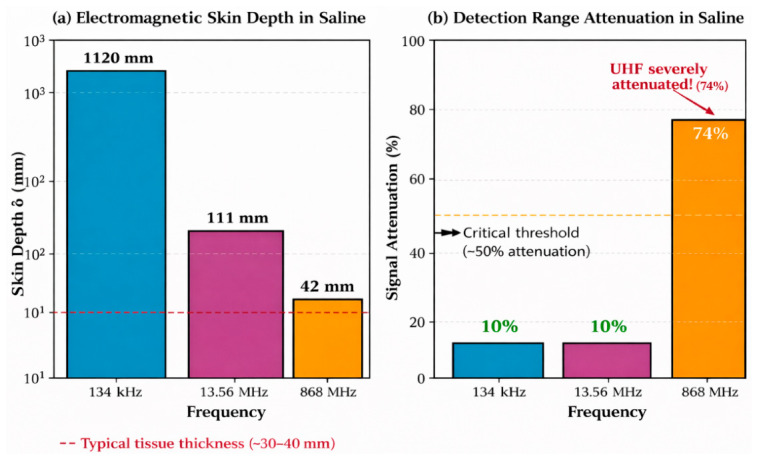
Tissue attenuation analysis: (**a**) electromagnetic skin depth in saline showing an order-of-magnitude reduction from LF to UHF; (**b**) detection range attenuation percentage revealing the critical 74% loss at UHF versus only 10% at LF/HF.

Figure 10 illustrates the relationship between skin depth and signal attenuation across frequency bands, demonstrating the physical basis for observed performance differences.

At a low frequency (134 kHz), electromagnetic coupling occurs exclusively through magnetic induction in the reactive near field. The exceptionally large skin depth (δ = 1.12 m in physiological saline) ensures minimal conductive tissue losses across all clinically relevant detection distances. This band achieved the highest through-tissue detection range (22.5 mm in saline), confirming its suitability for implanted transponder applications. However, the requirement for relatively large antenna structures (minimum practical diameter ~10 mm) and low data transfer rates (4 kbps) constrain miniaturised surgical probe integration.

The high-frequency band (13.56 MHz) maintains predominantly near-field magnetic coupling while enabling substantial antenna miniaturisation through ferrite core enhancement. The skin depth (δ = 111 mm) remains substantially larger than typical colorectal tissue thicknesses, explaining the identical 10% attenuation observed at LF. The 92% range preservation (20.7 mm vs. 22.5 mm) combined with 50% antenna diameter reduction represents a desirable engineering compromise. Additionally, the mature NFC commercial ecosystem at 13.56 MHz provides standardised protocols, certified reader ICs, and regulatory precedent that reduce development risk.

Ultra-high-frequency (868 MHz) operation introduces a fundamentally different electromagnetic regime characterised by radiative propagation and backscatter communication. While this enables dramatically extended free-space detection (68 mm, 2.7× improvement over LF) and the smallest transponder form factors (2.0 × 1.25 mm), the skin depth (δ = 42 mm) becomes comparable to tissue thicknesses. The resulting 74% attenuation produces inferior through-tissue performance (18 mm) despite superior capability in air—an inversion of the performance hierarchy that represents a critical finding for frequency selection.

The ferrite core enhancement at HF enables antenna miniaturisation through a well-understood physical mechanism: the NiZn ferrite rod (initial permeability μi = 125) concentrates magnetic flux within its volume, increasing the effective permeability from μr = 1 (air core) to μeff ≈ 10.7 (accounting for the demagnetisation factor N ≈ 0.1, Equation (7)). This flux concentration produces equivalent magnetic field intensity from a physically smaller structure, enabling the 50% diameter reduction (from 10 mm to 5 mm) while preserving 92% of the LF detection range (20.7 mm vs. 22.5 mm). The mature NFC ecosystem at 13.56 MHz—including standardised protocols (ISO 14443/15693 [60,61], NFC Forum), certified reader ICs (e.g., ST25R3916B), and extensive application support infrastructure—provides a development platform that significantly reduces engineering risk, regulatory complexity, and time-to-market for clinical system development.

#### 5.1.2. Quantitative Comparison

Table 11 presents a weighted multi-criteria analysis incorporating key parameters relevant to clinical system development. Criterion weights were derived through a simplified Delphi process involving three senior colorectal surgeons and two biomedical engineers. Panellists independently assigned importance weights (summing to 100%) across seven criteria, followed by a single reconciliation round to resolve discrepancies exceeding ±5%. The final weights reflect panel consensus, with tissue detection range receiving the highest weight (25%) as the primary functional requirement and antenna miniaturisation (20%) reflecting the critical constraint of laparoscopic instrument compatibility. Individual criterion scores (1–10 scale) were assigned based on quantitative experimental data where available and semi-quantitative assessment for less directly measurable attributes.

Sensitivity analysis: To assess ranking robustness, a perturbation analysis varied each criterion weight by ±5 percentage points (redistributed proportionally among remaining criteria). The 13.56 MHz band maintained the highest composite score across all 14 perturbation scenarios, with weighted scores ranging from 7.72 to 8.28. The ranking order (HF > LF > UHF) was preserved in 12 of 14 scenarios; in the remaining two (tissue range weight +5% or antenna miniaturisation weight −5%), LF and HF achieved near-identical scores (difference < 0.15 points) without reversing the HF preference. UHF consistently ranked third regardless of weight perturbation.

#### 5.1.3. Electromagnetic Basis for Tissue Attenuation Differences

The dramatic difference in tissue attenuation between LF/HF (10%) and UHF (74%) is explained by two compounding electromagnetic mechanisms. Power attenuation in conducting media follows P(z)/P_0_ = e^(−2z/δ)^. For propagation distance z = 20 mm through saline: at 134 kHz (δ = 1120 mm), P/P_0_ = 0.965 (3.5% power loss); at 13.56 MHz (δ = 111 mm), P/P_0_ = 0.698 (30% power loss); at 868 MHz (δ = 42 mm), P/P_0_ = 0.387 (61% power loss). The theoretical calculations align qualitatively with measured range reductions, though the relationship is non-linear due to the tag activation threshold characteristic.

Additionally, at UHF the impedance mismatch at tissue interfaces creates reflection losses absent in the quasi-static LF/HF regime. For saline (ε_c_ = 78 − j31 at 868 MHz), |Γ|^2^ ≈ 0.6, indicating that approximately 60% of incident power is reflected at the air–tissue interface before propagation losses occur. This mechanism substantially compounds skin-depth attenuation at UHF.

#### 5.1.4. Frequency Selection Recommendation

Based on the comprehensive quantitative and qualitative analysis, the 13.56 MHz high-frequency band is recommended as the optimal choice for next-generation RFID-based colorectal tumour localisation systems. This recommendation is predicated on the following convergent factors:

1. Adequate tissue penetration: The 20.7 mm detection range in saline encompasses most clinically relevant scenarios for colorectal surgery, where the typical tissue thickness between serosal surface and submucosal tag position ranges from 15 to 30 mm depending on anatomical location and patient habitus.
2. Superior miniaturisation: The 5 mm antenna diameter enables integration within standard laparoscopic instrument shafts (typically 5–12 mm diameter), supporting ergonomic probe designs compatible with existing surgical workflows.
3. NFC ecosystem leverage: The 13.56 MHz frequency corresponds to the global NFC standard (ISO 14443/15693, NFC Forum), providing access to mature development platforms (e.g., ST25R3916B evaluation kit), certified reader ICs with integrated analog front ends, and extensive application support infrastructure.
4. Regulatory pathway clarity: HF RFID and NFC technologies have established regulatory precedent in implantable medical devices, including passive sensing transponders [62] and monitoring implants and drug delivery systems, facilitating FDA 510(k) and EU MDR conformity assessment processes.

The 134 kHz LF band remains a viable alternative where maximum detection range is the overriding priority, or where regulatory alignment with existing FDA-cleared LF devices (LOCalizer) is strategically advantageous. The 868 MHz UHF band is not recommended for through-tissue detection of implanted transponders due to excessive tissue attenuation, though it may find application in surface-adjacent marking or hybrid multi-frequency architectures.

### 5.2. Angular Orientation Sensitivity and Mitigation Strategies

#### 5.2.1. Physical Basis for Angular Null Zones

The pronounced detection range degradation at orthogonal transponder orientation (θ_t_ = 90°) represents a fundamental consequence of the magnetic dipole coupling mechanism operative in near-field RFID systems. The mutual inductance *M* between reader antenna (coil 1) and tag antenna (coil 2) is as follows:

(10) $$M=k\sqrt{L_{1}L_{2}}=\frac{\mu_{n}N_{1}N_{2}A_{1}A_{2}}{2\pi r^{3}}\times cos(\theta )$$

where *k* is the coupling coefficient, *L*_1_ and *L*_2_ are coil inductances, *A*_1_ and *A*_2_ are effective coil areas, *r* is separation distance, and θ is the angle between coil axes. The cos(θ) dependence produces zero coupling at θ = 90°, creating the theoretical null zone.

In practice, the null zone is not absolute due to several secondary coupling mechanisms: (1) electric field coupling contributing at higher frequencies; (2) non-ideal coil geometry producing finite axial field extent; (3) ferrite core flux spreading in HF antennas. These effects explain the non-zero detection ranges (5.8–14.3 mm) measured at θ_t_ = 90° across all frequency bands. Nevertheless, the 64–79% range reduction relative to optimal alignment represents a substantial clinical constraint.

#### 5.2.2. Clinical Implications

The angular dependence of detection range introduces several clinical workflow considerations that must be addressed in system design and surgical protocol development:

1. Tag deployment orientation: Endoscopically placed transponders may assume unpredictable orientations within the submucosal tissue plane, potentially rotating between deployment and subsequent surgical procedure. If the tag axis aligns orthogonally to the anticipated surgical approach vector, detection failure may occur despite the tag being within nominal range. This uncertainty argues for either (a) orientation-controlled deployment techniques ensuring predictable tag alignment, or (b) reader systems capable of multi-axis interrogation.
2. Surgical approach variability: Laparoscopic colorectal procedures involve dynamic probe trajectories as the surgeon navigates around anatomical structures. A tag orientation optimal for one approach angle may present null-zone alignment from another direction. Surgical training must emphasise systematic probe manipulation protocols that sample multiple interrogation angles before concluding detection failure.
3. False negative risk: The clinical consequence of detection failure is surgical error-either unnecessary extended resection or, more seriously, inadequate margin achievement. The angular sensitivity therefore represents a patient safety consideration requiring robust mitigation.

#### 5.2.3. Technical Mitigation Approaches

Several engineering solutions can address angular sensitivity (Table 12):

(a) Orthogonal dual-axis antenna arrays: Incorporating two reader coils with perpendicular axes ensures that at least one coil maintains favourable coupling regardless of tag orientation. The detection criterion becomes a logical OR of the two channels, eliminating the null zone at the cost of increased probe complexity and diameter. Preliminary calculations suggest a dual-axis configuration with 3 mm diameter coils could fit within a 7 mm probe shaft.

(b) Three-axis antenna systems: Full orientation independence requires three orthogonal coils sampling all spatial directions. This approach additionally enables determination of tag orientation through relative signal strength analysis, supporting enhanced localisation algorithms. Implementation within laparoscopic dimensional constraints presents significant engineering challenges but may be feasible for robot-assisted platforms with relaxed probe size requirements.

(c) Rotating magnetic field: Phase-shifted excitation of orthogonal coils can generate a rotating magnetic field vector that sweeps through all orientations during each interrogation cycle. This approach automatically samples all angular configurations without requiring physical probe manipulation.

(d) Multi-tag deployment: Placing multiple transponders at different orientations around the tumour perimeter increases the probability that at least one tag presents favourable alignment. This clinical protocol approach transfers the redundancy from the reader to the tag side, potentially simplifying probe design while increasing procedure complexity and cost.

#### 5.2.4. Clinical Workflow Integration Considerations

Integration of an RFID-based localisation system into standard laparoscopic workflow requires addressing several practical considerations beyond electromagnetic performance. First, the reader probe must be compatible with standard sterilisation processes (autoclave at 134 °C or ethylene oxide); the ferrite-core HF antenna materials (NiZn ferrite, copper winding, and glass-encapsulated transponder) are inherently compatible, though connector and electronic interfaces require appropriate hermetic sealing. The glass-encapsulated transponder form factor has established biocompatibility per ISO 10993 series standards, as demonstrated by the LOCalizer system’s FDA clearance (K171067) and CE marking for long-term subcutaneous implantation [34]. Second, the system must provide real-time feedback with sufficient precision for surgical decision-making: the current system provides binary detection (tag present/absent within range) with distance estimation based on signal strength, which supports identification of the marked region but does not yet provide three-dimensional localisation accuracy for precision margin assessment. Third, electromagnetic compatibility with the surgical environment—including electrosurgical devices, metallic instruments, and imaging equipment—has not been characterised and represents a critical requirement for clinical translation. Finally, the system should provide intuitive audiovisual feedback interpretable without requiring the surgeon to divert attention from the operative field, following established ergonomic principles from existing surgical navigation platforms.

### 5.3. Comparative Analysis with Existing Technologies

The experimental findings enable direct comparison with commercially available RFID-based surgical localisation systems, contextualising the achieved performance within the existing technology landscape (Table 13).

LOCalizer (Hologic): This FDA-cleared system employs 134 kHz LF technology for breast lesion localisation. Published clinical data report detection ranges of 20–30 mm with >97% localisation success rates in prospective series. The measured 22.5 mm saline detection range in this study aligns well with LOCalizer performance, validating both the experimental methodology and the clinical viability of similar LF designs for colorectal application. The LOCalizer transponder (12 mm × 2 mm glass capsule) presents a biocompatible form factor directly transferable to colorectal deployment.

SAVI SCOUT (Cianna Medical): Operating at 915 MHz UHF, SAVI SCOUT reports detection ranges up to 60 mm. However, this specification likely represents performance in air rather than through-tissue capability. The measured 74% tissue attenuation at UHF suggests that SAVI SCOUT’s clinical effectiveness relies on the relatively superficial positioning of breast lesions (typically < 20 mm from skin surface) rather than demonstrated deep-tissue penetration. Direct translation of this UHF approach to colorectal applications—where detection through 20–30 mm of bowel wall and perirectal fat is required—appears problematic based on the experimental evidence.

#### Comparison with Recent Literature (2024–2026)

Table 14 provides a contextual comparison with recent wireless surgical localisation studies, positioning the current findings within the evolving technology landscape.

It should be noted that direct comparison across studies is limited by differences in validation medium, detection criterion definition, and system configuration. The present study is the first to provide systematic tri-frequency characterisation specifically for the colorectal application, addressing a knowledge gap where all prior RFID localisation work has focused on breast surgery.

### 5.4. Limitations

#### 5.4.1. Methodological Limitations

Tissue-equivalent medium simplification: Homogeneous saline solution (0.9% NaCl), while providing standardised conductivity comparable to average tissue values, does not replicate the layered dielectric structure of colorectal anatomy. The heterogeneous tissue composition—with high-permittivity mucosa, intermediate muscle, and low-permittivity fat—creates impedance discontinuities and wave refraction phenomena absent in homogeneous saline. Detection performance in clinical scenarios may therefore differ from saline measurements, potentially favourably (if fat layers dominate) or unfavourably (if muscle/mucosa predominate). Specifically, the four-layer colorectal wall presents three dielectric interfaces: mucosa to submucosa (Δεr ≈ 60), submucosa to muscularis (Δεr ≈ 20), and muscularis to fat (Δεr ≈ 42 at 868 MHz), each producing partial wave reflection at UHF. At LF/HF, where the quasi-static approximation holds, these permittivity contrasts have negligible effect on magnetic field coupling, and the attenuation is governed primarily by the volume-weighted average conductivity. The saline conductivity (σ = 1.5 S/m) substantially exceeds this weighted average (~0.3–0.5 S/m when mesorectal fat comprising 30–60% of path length is included), confirming that saline measurements represent a conservative upper bound for tissue attenuation at LF/HF. At UHF, the layered geometry introduces interface reflections that may either increase or decrease net attenuation relative to homogeneous saline. Additionally, the absence of tissue perfusion, bowel motility, and gas-containing lumen eliminates dynamic effects that may influence detection reliability in vivo. Future studies should employ multi-layer phantoms with frequency-dependent dielectric properties [63].

Static measurement conditions: All measurements were performed under static conditions with a controlled probe–tag geometry. Clinical surgery involves dynamic probe manipulation, tissue deformation, respiratory motion, and variable approach angles. The static angular characterisation provides necessary but not sufficient data for predicting clinical detection reliability.

Angular resolution: The 30° angular increment employed in the measurement matrix may miss fine-structure variations in the detection envelope. While computationally validated models suggest smooth angular transitions, experimental confirmation at higher resolution would strengthen conclusions regarding null-zone extent.

Single transponder type per frequency: Each frequency band was characterised with a single transponder model (EM4100 at 134 kHz, NTAG216 at 13.56 MHz, Murata at 868 MHz). Performance with alternative transponders may differ due to variations in antenna Q-factor, IC sensitivity, and form factor.

#### 5.4.2. Technical Limitations

UHF antenna dimensions: The near-field UHF antenna (60 × 60 mm) substantially exceeds dimensional constraints for laparoscopic probe integration. While representing commercially available near-field UHF technology, this configuration does not demonstrate clinical feasibility. Further miniaturisation-potentially at the cost of reduced detection range-would be required for practical implementation.

Binary detection criterion: The experimental protocol employed binary pass/fail detection based on protocol acknowledgment. This approach does not capture quantitative signal quality metrics (RSSI, BER, read rate) that could inform more sophisticated detection algorithms or provide confidence measures for clinical decision-making.

Absence of EMC characterisation: Electromagnetic compatibility with surgical environment equipment (electrosurgery, monitors, imaging) was not evaluated. The controlled laboratory environment does not reproduce the electromagnetic noise floor of a typical operating theatre.

Long-term stability: Transponder performance stability over extended implantation periods (days to weeks) was not assessed. Biocompatibility-related effects (encapsulation, corrosion) could potentially degrade detection performance over time.

#### 5.4.3. Preclinical Scope

The present study is preclinical in nature, comprising exclusively computational simulations and experimental phantom measurements under controlled laboratory conditions. The results establish electromagnetic feasibility and frequency selection guidance but do not constitute evidence of clinical safety or efficacy. Translation to clinical practice requires sequential validation through multi-layer tissue phantoms, ex vivo animal tissue testing, in vivo large animal models, and ultimately prospective human feasibility studies under appropriate regulatory oversight. The current conclusions should be interpreted as foundational engineering evidence supporting clinical system development, not as indicators of immediate clinical applicability. The regulatory pathway for the proposed HF system would likely follow FDA 510(k) clearance with the LOCalizer system (K171067) as predicate device, or EU MDR conformity assessment under Class IIb implantable device classification, leveraging the established regulatory precedent of NFC-based implantable medical devices [62].

### 5.5. Future Research Directions

Multi-layer tissue phantom development: Construction of anatomically realistic colorectal phantoms incorporating distinct mucosa, submucosa, muscularis, and fat layers with frequency-dependent dielectric properties. Such phantoms would enable refined detection range predictions and support standardised inter-laboratory comparison.

Dual-axis antenna optimisation: Design and fabrication of orthogonal dual-coil HF antenna arrays achieving orientation-independent detection within laparoscopic dimensional constraints (<8 mm diameter). Optimisation targets include balanced sensitivity across orientations, minimal cross-coupling between axes, and integration with standard instrument interfaces.

EMC assessment: Systematic characterisation of RFID reader performance in the presence of electrosurgical devices, metallic instruments, and imaging equipment representative of the surgical environment.

Sterilizable instrument integration: Development of reader antenna configurations compatible with standard sterilisation processes (autoclave and ethylene oxide) and established laparoscopic instrument manufacturing practices.

## 6. Conclusions

This preclinical paper has presented an electromagnetic characterisation of RFID technology for intraoperative tumour localisation in minimally invasive colorectal surgery. Computational modelling and systematic experimental validation across three frequency bands—134 kHz, 13.56 MHz, and 868 MHz—established fundamental performance parameters, identified critical design trade-offs, and provided evidence-based guidance for clinical system development.

The experimental characterisation yielded several findings with direct implications for surgical RFID system design:

(1) Frequency-dependent tissue attenuation: Maximum detection distances in air were 25.0 mm (LF), 23.0 mm (HF), and 68.0 mm (UHF). In physiological saline, this hierarchy inverted: 22.5 mm (LF), 20.7 mm (HF), and 18.0 mm (UHF), with tissue attenuation of 10% at LF/HF versus 74% at UHF. This finding—that UHF achieves superior air performance but inferior tissue penetration—represents a critical insight for implanted transponder applications, attributable to the compounding effects of reduced skin depth (δ = 42 mm at UHF) and impedance mismatch reflection losses (|Γ|^2^ ≈ 0.6).

(2) Angular orientation sensitivity: Characterisation across 28 angular configurations revealed pronounced null zones at orthogonal transponder alignment (θ_t_ = 90°), with range reductions of 64% (LF), 70% (HF), and 79% (UHF). This orientation dependence, inherent to the cos(θ) coupling mechanism, represents a fundamental constraint requiring multi-axis antenna mitigation strategies. The progressive increase in null-zone severity from LF (64%) through HF (70%) to UHF (79%) reflects the increasingly radiative nature of electromagnetic propagation with frequency: at UHF, the electric field polarisation sensitivity compounds the fundamental cos(θ) magnetic coupling dependence, producing more severe orientation-dependent losses.

(3) Antenna miniaturisation: The 13.56 MHz system achieved 92% of LF detection range while enabling 50% antenna diameter reduction (5 mm vs. 10 mm) through ferrite core flux concentration, supporting integration within standard laparoscopic instrument dimensions (5–12 mm).

(4) Computational model validation: Agreement between FEM predictions and experimental measurements (r^2^ = 0.975, RMSE = 2.01 mm, *N* = 154, near-unity regression slope with negligible bias) validated the modelling methodology across all frequency bands, establishing a reliable predictive framework for continued antenna optimisation.

The validation metrics warrant explicit interpretation; r^2^ = 0.975 indicates that the FEM models explain 97.5% of the observed variance in detection range, with only 2.5% residual variability. The RMSE of 2.01 mm falls below the clinically relevant resolution threshold (surgical margins are typically assessed at 5–10 mm scale), confirming that model prediction uncertainty is smaller than the surgical decision tolerance. The near-unity regression slope (1.001 ± 0.025) and near-zero intercept (0.02 ± 0.56 mm) confirm the absence of systematic proportional or offset bias, establishing the FEM framework as a reliable and unbiased predictive tool for continued antenna optimisation and clinical system design.

Based on a weighted multi-criteria analysis incorporating expert panel input and verified through sensitivity testing, the 13.56 MHz band emerges as the preferred candidate for clinical development. This assessment reflects the convergence of adequate tissue penetration, superior miniaturisation potential, higher data rates (26–424 kbps versus 4 kbps at LF), and the mature NFC ecosystem (ISO 14443/15693) that reduces development risk.

The principal contributions of this study include the first systematic tri-frequency comparison for colorectal surgical localisation under standardised conditions, quantitative tissue attenuation characterisation with frequency-dependent uncertainty analysis, comprehensive angular sensitivity mapping, and experimentally validated computational models applicable to continued medical device design.

Critical challenges for clinical translation include angular null-zone mitigation through orthogonal dual-axis antenna arrays, development of three-dimensional localisation algorithms, electromagnetic compatibility validation with surgical environment equipment, and systematic pre-clinical evaluation in multi-layer tissue phantoms [63] and large animal models preceding first-in-human feasibility studies.

This study establishes the electromagnetic evidence base upon which these subsequent development steps can proceed with quantified performance expectations and informed frequency selection.

This study demonstrates that passive RFID technology provides a technically viable foundation for next-generation intraoperative tumour localisation, addressing critical limitations of current tattooing-based methods. While engineering challenges remain—particularly regarding angular sensitivity mitigation and three-dimensional localisation capability—the results establish the electromagnetic basis for clinical system development with the potential to improve surgical precision in minimally invasive colorectal cancer surgery.

## Figures and Tables

**Table 1:** RFID system configurations for tri-frequency comparative evaluation.

Parameter	134 kHz (LF)	13.56 MHz (HF)	868 MHz (UHF)
Antenna type	Air-core solenoid	Ferrite rod (NiZn)	Near-field loop
Antenna size	Ø10 × 10 mm	Ø5 × 15 mm	60 × 60 mm
Transponder	EM4100 glass tag	NTAG216 glass	Murata UHF chip
Tag size	Ø2 × 12 mm	Ø2 × 12 mm	2.0 × 1.25 mm
Reader platform	Custom (MCU)	ST STEVAL-25R3916B	Impinj R420

**Table 2:** Solver configurations for tri-frequency FEM simulations.

Parameter	134 kHz	13.56 MHz	868 MHz
Platform	FEMM 4.2	COMSOL 6.2, AC/DC	COMSOL 6.2, RF Module
Physics	Magnetic Fields (mf)	Magnetic Fields (mf)	EM Waves, Freq. Domain (emw)
Geometry	2D axisymmetric	2D axisymmetric/3D	3D
Element type	1st-order triangular	2nd-order triangular	2nd-order curl
Solver	Newton-Raphson (10^−8^)	MUMPS direct (10^−6^)	PARDISO iterative
Boundary	Asymptotic (r → ∞)	Infinite element	PML (λ/4 thickness)
Mesh size	45–65 k elements	80–120 k elements	1.5–2.5 M elements

**Table 3:** Reader antenna specifications for 134 kHz FEM model.

Parameter	Value
Coil outer diameter	10 mm
Coil length	10 mm
Wire diameter	0.08 mm (AWG 40)
Number of turns	1000
Winding layers	~25 (calculated)
Calculated inductance	~700 µH
Excitation current	100 mA (peak)
Core material	Air (μr = 1)

**Table 4:** Material electromagnetic properties at 134 kHz.

Material	μr	σ (S/m)	εr
Air	1.0	0	1.0
Copper (wire)	0.999991	5.8 × 10^7^	1.0
Saline (0.9%)	1.0	1.5	~80
Muscle tissue	1.0	0.4	~10,000
Fat tissue	1.0	0.02	~1500

**Table 5:** Ferrite-core antenna geometry specifications for 13.56 MHz simulations.

Parameter	Open Rod	Shielded Rod	Pot Core
Ferrite type	NiZn	NiZn	NiZn
Initial permeability μi	125	125	125
Core dimensions	Ø5 × 15 mm	Ø5 × 15 mm	Ø10 × 5 mm
Winding turns	10	10	15
Calculated inductance	~800 nH	~750 nH	~1.2 µH
Shield material	None	Copper	Ferrite

**Table 6:** Material electromagnetic properties at 13.56 MHz.

Material	μ_r_	ε_r_	σ (S/m)	tan δ
Air	1.0	1.0	0	0
NiZn ferrite	125 − j2.5	12	10^−5^	0.02
Copper	1.0	1.0	5.8 × 10^7^	—
Saline (0.9%)	1.0	78	1.5	2.3
Muscle	1.0	138	0.6	0.6
Fat	1.0	12	0.03	0.3

**Table 7:** Near-field UHF antenna specifications for 868 MHz simulations.

Parameter	Value
Antenna type	Segmented loop (4 segments)
Overall dimensions	60 × 60 mm
Trace width	3 mm
Substrate	FR-4 (ε_r_ = 4.4, tan δ = 0.02)
Substrate thickness	1.6 mm
Copper thickness	35 µm
Ground plane	Partial (50 × 50 mm, rear)
Feed	Lumped port (50 Ω)
Input power	100 mW (20 dBm)

**Table 8:** Tissue dielectric properties at 868 MHz (IT’IS Foundation): δ = skin depth, and t = typical thickness [59].

Tissue	εr′	εr″	σ (S/m)	δ (mm)	t (mm)
Skin	41.3	17.8	0.87	48	1–2
Fat	5.5	1.0	0.05	220	10–30
Muscle	54.8	19.4	0.95	43	2–3
Mucosa	62.2	21.3	1.04	40	0.5–1
Saline	78.0	30.7	1.50	42	—

**Table 9:** Comprehensive cross-frequency performance comparison.

Parameter	134 kHz	13.56 MHz	868 MHz
Max range (air)	25.0 ± 0.9 mm	23.0 ± 1.1 mm	68.0 ± 2.3 mm
Max range (saline)	22.5 ± 1.0 mm	20.7 ± 1.2 mm	18.0 ± 1.4 mm
Tissue attenuation	10%	10%	74%
Skin depth (saline)	1.12 m	111 mm	42 mm
Null-zone reduction	64%	70%	79%
Antenna diameter	10 mm	5 mm	60 mm
Data rate	4 kbps	26–424 kbps	40–640 kbps
Failed configs (saline)	2/28	3/28	8/28

**Table 10:** Computational model validation metrics stratified by frequency band. CI: confidence interval; RMSE: root mean square error; LoA: Bland–Altman 95% limits of agreement.

Validation Metric	134 kHz	13.56 MHz	868 MHz	All bands
N (paired observations)	56	56	42	154
r^2^ (Pearson)	0.973	0.969	0.968	0.975
Slope (95% CI)	0.983 ± 0.045	0.932 ± 0.046	1.003 ± 0.058	1.001 ± 0.025
Intercept, mm (95% CI)	0.20 ± 0.76	0.82 ± 0.66	0.29 ± 1.92	0.02 ± 0.56
RMSE (mm)	1.00	1.13	3.43	2.01
Mean bias (mm)	−0.06	−0.08	+0.36	+0.05
Mean|error|(mm)	0.86	0.98	2.71	1.41
Max|error|(mm)	1.90	2.10	7.40	7.40
Max relative error (%)	±8.2	±10.0	±12.1	±12.1
Mean relative error (%)	5.4	7.5	9.4	7.3
LoA lower bound (mm)	−2.03	−2.31	−6.40	−3.90
LoA upper bound (mm)	+1.91	+2.15	+7.12	+3.99

**Table 11:** Weighted multi-criteria analysis for frequency band selection (scores 1–10, higher = better).

Criterion	Weight	134 kHz	13.56 MHz	868 MHz	Rationale
Tissue detection range	25%	10	9	7	Primary function
Antenna miniaturisation	20%	5	9	3	Probe integration
Tag miniaturisation	15%	6	6	10	Implantability
Data rate capability	10%	3	8	9	Sensor integration
Ecosystem maturity	10%	7	10	8	Development ease
Regulatory precedent	10%	9	8	7	Approval pathway
Angular tolerance	10%	7	6	4	Clinical robustness
Weighted Score	100%	7.15	8.00	6.35	-

**Table 12:** Comparison of angular sensitivity mitigation approaches.

Mitigation Approach	Effectiveness	Probe Diameter	Complexity	Cost Impact
Dual-axis array	High (>90%)	7–8 mm	Moderate	+30%
Three-axis system	Complete	10–12 mm	High	+60%
Rotating field	High (>95%)	8–10 mm	High	+50%
Multi-tag protocol	Moderate-High	5 mm	Low	+100% tags

**Table 13:** Comparison with commercial RFID surgical localisation systems (* Estimated based on experimental tissue attenuation data).

Parameter	LOCalizer	SAVI SCOUT	This Study (HF)	Target
Frequency	134 kHz	915 MHz	13.56 MHz	-
Detection range (air)	30–40 mm	60 mm	23 mm	>20 mm
Detection range (tissue)	20–30 mm	~15–20 mm *	20.7 mm	>15 mm
Tag size	12 × 2 mm	12 mm reflector	12 × 2 mm	<15 mm
Probe diameter	~15 mm	~20 mm	5 mm (target)	<12 mm
Regulatory status	FDA cleared	FDA cleared	Investigational	-

**Table 14:** Comparison with recent wireless surgical localisation studies (2024–2026). Detection ranges are reported as maximum values under stated conditions.

Study	Year	Technology/Frequency	Application	Detection Range	Validation Medium	Key Finding
**This study (HF)**	2026	Passive RFID, 13.56 MHz	Colorectal (phantom)	20.7 ± 1.2 mm (saline)	0.9% NaCl saline	Optimal frequency for colorectal; 5 mm antenna diameter
**This study (LF)**	2026	Passive RFID, 134 kHz	Colorectal (phantom)	22.5 ± 1.0 mm (saline)	0.9% NaCl saline	Maximum tissue penetration range; 10% tissue attenuation
**This study (UHF)**	2026	Passive RFID, 868 MHz	Colorectal (phantom)	18.0 ± 1.4 mm (saline)	0.9% NaCl saline	74% tissue attenuation; inferior through-tissue performance
**Daly et al. **[19]	2025	RFID (LOCalizer), 134 kHz	Breast (clinical)	20–30 mm (tissue)	In vivo (multi-centre)	Systematic review and meta-analysis; >95% localisation accuracy
**Sanli et al. **[37]	2024	RFID (LOCalizer) vs. Magseed (magnetic)	Breast (phantom)	Not separately reported	Turkey breast model	100% localisation success both methods; equivalent surgical margins
**Almalki et al. **[22]	2023	RFID (LOCalizer), 134 kHz	Breast (clinical)	~25 mm (tissue)	In vivo (258 patients)	>95% accuracy within 10 mm NHS standard
**Magseed pooled meta-analysis **[38]	2026	Magnetic seed (passive, Endomag)	Breast (clinical)	~30 mm (probe distance)	In vivo (2117 patients)	7.6% positive margin rate; ~100% retrieval success
**Zou et al. **[33]	2025	NFC/RFID review (13.56 MHz focus)	Implantable devices (review)	Variable (application-dependent)	Various (in vivo, phantom)	NFC ecosystem mature for implantable biomedical sensing

## Data Availability

The data presented in this study are available in Supplementary Materials.

## Supplementary Materials

The following supporting information can be downloaded at: https://www.mdpi.com/article/10.3390/diagnostics16091318/s1, Table S1: Detection range measurements (mm) across 28 angular configurations for three RFID frequency bands in air and physiological saline (0.9% NaCl, σ ≈ 1.5 S/m at 25 °C), Table S2: Summary statistics of detection range measurements across 28 angular configurations.
