# Supplementary material for: RFID Technology for Intraoperative Localisation of Small Colorectal Tumours: Electromagnetic Analysis and Experimental Validation
Source: Diagnostics (Basel). 2026 Apr 28;16(9):1318. doi: 10.3390/diagnostics16091318 (PMC13162962; doi:10.3390/diagnostics16091318)
Supplement: Supplementary file 1 [file diagnostics-16-01318-s001.zip › diagnostics-4246146-supplementary.pdf]

Supplementary Table S1. Detection range measurements (mm) across 28 angular configurations for three RFID frequency bands in air and physiological saline (0.9% NaCl, σ ≈ 1.5 S/m at 25°C). Values represent mean ± expanded uncertainty (k = 2, 95% confidence level). Dash (—) indicates detection failure defined as inability to achieve consistent packet reception across five consecutive interrogation cycles at any separation distance ≤ 70 mm.

		134 kHz	134 kHz	13.56 MHz	13.56 MHz	868 MHz	868 MHz
**θa (°)**	**θt (°)**	**Air (mm)**	**Saline (mm)**	**Air (mm)**	**Saline (mm)**	**Air (mm)**	**Saline (mm)**
-90	0	23.5 ± 0.9	20.8 ± 1.0	20.6 ± 1.1	18.2 ± 1.2	56.9 ± 2.3	13.6 ± 1.4
-90	30	20.1 ± 0.9	17.9 ± 1.0	17.1 ± 1.2	15.1 ± 1.3	44.9 ± 2.5	9.1 ± 1.5
-90	60	12.6 ± 1.0	11.1 ± 1.1	9.7 ± 1.2	8.3 ± 1.4	21.8 ± 2.8	—
-90	90	8.5 ± 1.0	7.6 ± 1.2	5.8 ± 1.4	4.8 ± 1.5	12.0 ± 3.2	—
-60	0	23.7 ± 0.9	21.3 ± 1.0	21.1 ± 1.1	18.9 ± 1.2	59.0 ± 2.3	14.0 ± 1.4
-60	30	20.2 ± 0.9	17.8 ± 1.0	17.3 ± 1.2	15.2 ± 1.3	46.5 ± 2.5	9.5 ± 1.5
-60	60	12.7 ± 1.0	10.9 ± 1.1	9.8 ± 1.2	8.3 ± 1.4	22.7 ± 2.8	—
-60	90	8.3 ± 1.0	7.3 ± 1.2	6.2 ± 1.4	5.4 ± 1.5	12.2 ± 3.2	—
-30	0	24.2 ± 0.9	21.7 ± 1.0	22.0 ± 1.1	19.8 ± 1.2	63.8 ± 2.3	15.9 ± 1.4
-30	30	20.7 ± 0.9	18.2 ± 1.0	18.0 ± 1.2	16.0 ± 1.3	49.9 ± 2.5	10.9 ± 1.5
-30	60	13.3 ± 1.0	11.5 ± 1.1	10.4 ± 1.2	8.9 ± 1.4	24.3 ± 2.8	4.5 ± 1.7
-30	90	8.6 ± 1.0	7.4 ± 1.2	6.6 ± 1.4	5.2 ± 1.5	13.3 ± 3.2	—
0	0	25.0 ± 0.9	22.5 ± 1.0	23.0 ± 1.1	20.7 ± 1.2	68.0 ± 2.3	18.0 ± 1.4
0	30	21.2 ± 0.9	19.0 ± 1.0	19.2 ± 1.2	16.9 ± 1.3	53.5 ± 2.5	12.4 ± 1.5
0	60	13.5 ± 1.0	11.9 ± 1.1	10.8 ± 1.2	9.3 ± 1.4	26.0 ± 2.8	5.5 ± 1.7
0	90	9.0 ± 1.0	7.9 ± 1.2	6.9 ± 1.4	5.8 ± 1.5	14.3 ± 3.2	—
+30	0	24.5 ± 0.9	21.8 ± 1.0	22.1 ± 1.1	19.9 ± 1.2	64.0 ± 2.3	16.1 ± 1.4
+30	30	21.0 ± 0.9	18.3 ± 1.0	18.2 ± 1.2	16.2 ± 1.3	50.1 ± 2.5	11.0 ± 1.5
+30	60	13.5 ± 1.0	11.6 ± 1.1	10.5 ± 1.2	9.0 ± 1.4	24.4 ± 2.8	4.7 ± 1.7
+30	90	8.8 ± 1.0	7.6 ± 1.2	6.7 ± 1.4	5.4 ± 1.5	13.5 ± 3.2	—
+60	0	23.9 ± 0.9	21.4 ± 1.0	21.3 ± 1.1	19.1 ± 1.2	59.2 ± 2.3	14.1 ± 1.4
+60	30	20.3 ± 0.9	17.9 ± 1.0	17.5 ± 1.2	15.3 ± 1.3	46.6 ± 2.5	9.6 ± 1.5
+60	60	13.0 ± 1.0	11.1 ± 1.1	10.1 ± 1.2	8.5 ± 1.4	22.9 ± 2.8	—
+60	90	8.5 ± 1.0	7.4 ± 1.2	6.5 ± 1.4	5.5 ± 1.5	12.3 ± 3.2	—
+90	0	23.7 ± 0.9	20.9 ± 1.0	20.7 ± 1.1	18.4 ± 1.2	57.2 ± 2.3	13.8 ± 1.4
+90	30	20.2 ± 0.9	18.0 ± 1.0	17.4 ± 1.2	15.2 ± 1.3	45.1 ± 2.5	9.3 ± 1.5
+90	60	12.8 ± 1.0	11.2 ± 1.1	10.0 ± 1.2	8.4 ± 1.4	22.0 ± 2.8	—
+90	90	8.8 ± 1.0	7.8 ± 1.2	6.1 ± 1.4	5.0 ± 1.5	12.3 ± 3.2	—

Notes: θa = reader antenna rotation angle; θt = transponder tilt angle (0° = coaxial alignment, 90° = orthogonal). Measurement uncertainty propagated from positioning stage accuracy (±0.5 mm), step resolution (0.5 mm), and detection threshold transition width (0.3–0.5 mm for LF/HF, 0.8–1.2 mm for UHF). Due to the cylindrical symmetry of the antenna geometry, detection ranges at positive and negative reader angles (θa and −θa) exhibited <3% variation; both values are reported for completeness.

Supplementary Table S2. Summary statistics of detection range measurements across 28 angular configurations.

Statistic	134 kHz	134 kHz	13.56 MHz	13.56 MHz	868 MHz	868 MHz
	Air	Saline	Air	Saline	Air	Saline
Maximum (θt = 0°, θa = 0°)	25.0	22.5	23.0	20.7	68.0	18.0
Minimum (all configurations)	8.3	7.3	5.8	4.8	12.0	4.5
Mean ± SD (all 28 configs)	16.6 ± 6.1	14.6 ± 5.5	14.0 ± 6.0	12.2 ± 5.6	36.4 ± 19.4	11.3 ± 3.9*
Mean ± SD (θt = 0° only, n = 7)	24.1 ± 0.5	21.5 ± 0.6	21.5 ± 0.9	19.3 ± 0.9	61.2 ± 4.2	15.1 ± 1.6
Mean ± SD (θt = 90° only, n = 7)	8.6 ± 0.2	7.6 ± 0.2	6.4 ± 0.4	5.3 ± 0.3	12.8 ± 0.9	— [b]
Detection failures (saline)	0/28	0/28	0/28	0/28	0/28	11/28
Range ratio (θt = 90°/θt = 0°)	0.36	0.35	0.30	0.27	0.21	— [b]
Tissue attenuation at max range (%)	—	10.0	—	10.0	—	73.5
Mean tissue attenuation (%)	—	11.7	—	12.5	—	69.0

** Computed over 17 configurations with successful detection (11/28 detection failures in saline). [b] All seven θt = 90° configurations resulted in detection failure in saline at 868 MHz. Tissue attenuation at max range calculated as (Air_max − Saline_max)/Air_max × 100%.*
